# Transcutaneous electrical acupoint stimulation for upper limb motor recovery after stroke: a systematic review and meta-analysis

**DOI:** 10.3389/fnagi.2024.1438994

**Published:** 2024-11-27

**Authors:** Xiaoyu Wang, Lianjun Yin, Yikun Wang, Haining Zhang, Shiying Zhang, Jiantong Wu, Shun Fan, Zhengfei Li, Huanan Li, Jingui Wang

**Affiliations:** ^1^Department of Tuina, First Teaching Hospital of Tianjin University of Traditional Chinese Medicine, Tianjin, China; ^2^National Clinical Research Center for Chinese Medicine Acupuncture and Moxibustion, Tianjin, China; ^3^The Institute of Traditional Chinese Medicine Massage of Tianjin Health Commission, Tianjin, China; ^4^Rehabilitation Medicine, The Third Affiliated Hospital of Southern Medical University, Guangzhou, China

**Keywords:** transcutaneous electrical acupoint stimulation (TEAS), stroke, upper limb function, meta-analysis, systematic review

## Abstract

**Background:**

Transcutaneous electrical acupoint stimulation (TEAS) is an innovative, non-invasive therapy that stimulates the contraction of paralyzed muscles in the upper limbs, promoting functional recovery. Several studies have demonstrated the efficacy of TEAS in restoring upper limb function. This study aims to evaluate the impact of TEAS on upper limb motor recovery after stroke.

**Objectives:**

This study aims to evaluate the influence of TEAS on upper limb motor recovery after stroke and improve the quality of life in such patients.

**Methods:**

Eight databases were systematically searched from inception to 1st October 2024. Two independent reviewers conducted the screening and data extraction of the study. The primary outcome measure was the Fugl Meyer Assessment of the Upper Extremity (FMA-UE), which evaluates upper extremity motor function in stroke patients. Secondary outcomes included the Modified Ashworth Scale (MAS) for assessing spasticity and the Modified Barthel Index (MBI) to evaluate patients’ abilities to perform activities of daily living. Data synthesis was conducted using RevMan 5.4 and Stata 14.0. The GRADE method was employed to assess the quality of evidence.

**Results:**

A total of 16 trials involving 1,218 stroke patients were included in this meta-analysis. Meta-analysis showed that the TEAS significantly improved upper limb function (SMD = 1.70, 95CI% = 1.09 to 2.31, *p* < 0.00001, *I*^2^ = 93%; low certainty of evidence), reduced spasticity (SMD = −1.18, 95CI% = −1.79 to −0.58, *p* < 0.00001, *I*^2^ = 90%; very low certainty of evidence), and enhanced the ability to perform daily activities (SMD = 1.53, 95CI% = 0.85 to 2.20, *p* < 0.00001, *I*^2^ = 95%; low certainty of evidence).

**Conclusion:**

Our results indicated that TEAS improved motor function and functional activities and reduced muscle tone in the upper limbs after stroke. However, these results should be interpreted with caution due to the limited strength of the evidence. High-quality, larger sample, multi-center studies are needed to validate these preliminary findings.

**Systematic review registration:**

This study was registered on PROSPERO with registration number CRD42024592509. https://www.crd.york.ac.uk/prospero/display_record.php?ID=CRD42024592509

## 1 Introduction

Stroke is a condition characterized by damage to brain tissue caused by the rupture or obstruction of blood vessels within the brain (Sacco et al., 2013). According to the Global Burden of Disease Study (GBD), stroke ranks as the third leading cause of disability and mortality, accounting for 87.4 deaths (95% UI 79.5–94.4) per 100,000 population (GBD 2021 Causes of Death Collaborators, 2024). In China alone, more than 70 million individuals are affected by stroke, making it the leading cause of death in the country. Furthermore, as the population continues to age, the burden associated with stroke is expected to increase significantly (Tu and Wang, 2023; Wang et al., 2022).

After the onset of stroke, a variety of adverse manifestations can occur rapidly, most often accompanied by sensory and limb dysfunction, which severely impacts patients’ daily lives (Dawson et al., 2021). Studies have shown that approximately 55–75% of patients experience varying degrees of upper limb motor dysfunction (Ma et al., 2021). Relevant studies have demonstrated a strong correlation between upper limb motor function and activities of daily living (ADL) in stroke patients, which places a significant burden on caregivers, families, and society (Meng et al., 2022).

Upper limb motor dysfunction resulting from damage to upper motor neurons is characterized by poor motor ability, spasticity and flexion of the fingers, and impaired fine motor control of the hand (Zhou et al., 2021). Compared to lower limb motor dysfunction after a stroke, upper limb motor dysfunction is more difficult to recover from, with a higher incidence and a longer recovery period (Julie et al., 2017). Because fine motor tasks, especially those involving the hand, require precision, regaining normal function is challenging, and relying solely on traditional rehabilitation training often yields unsatisfactory outcomes.

Neurorehabilitation for upper limb dysfunction has become a central focus of post-stroke recovery efforts (Huang et al., 2024; Tang et al., 2024). Based on insights into brain functional remodeling, brain functional connectivity, cortical reorganization, and neural plasticity, various rehabilitation methods have been developed (Muller et al., 2024). Some of these methods, such as robotic exoskeleton training and constraint-induced movement therapy, have shown significant effectiveness (Zhang et al., 2022; Thrane et al., 2015).

Constraint-induced movement therapy, in particular, has shown positive rehabilitation outcomes for patients with acute or subacute strokes (Liu et al., 2017). However, for stroke survivors with severe motor deficits, the treatment outcomes are often minimal, and recovery remains unpredictable (Coscia et al., 2019).

Robotic training may be a viable alternative for patients with severe upper limb injuries, though the high cost of robotic devices poses a challenge (Zhang et al., 2022). Other therapeutic techniques, such as mirror therapy (Nogueira et al., 2021) and neurodevelopmental treatment (Langhammer and Stanghelle, 2011), appear to lack strong evidence supporting their effectiveness in improving upper limb motor function after a stroke. Therefore, new rehabilitation methods should be developed to enhance upper limb motor function impairment following a stroke (Pollock et al., 2014).

Electrical stimulation therapy for post-stroke spasticity has become a major area of interest. The American Heart Association guidelines for adult stroke rehabilitation endorse neuromuscular electrical stimulation as an effective approach for the transient alleviation of spasticity (Winstein et al., 2013). Transcutaneous electrical acupoint stimulation (TEAS) is a non-invasive stimulation therapy that combines the advantages of Chinese acupuncture and transcutaneous electrical nerve stimulation (Pan et al., 2023). TEAS uses low-frequency pulsed direct current to electrically stimulate peripheral acupoints and surrounding tissues. This stimulation facilitates the transmission of information to the central nervous system, thereby enhancing local neuromuscular function (Szmit et al., 2023).

TEAS mimics the effects of acupuncture on specific acupoints, stimulating muscle cell activity and facilitating the restoration of upper limb function and active control capabilities as swiftly as possible. This approach effectively alleviates symptoms of spasticity (Yan and Hui-Chan, 2009). Moreover, it activates relevant regions of the cerebral cortex, eliciting responses from neurons associated with the upper limb. This process is beneficial for enhancing muscle strength and improving hand coordination (Alwhaibi et al., 2021). Compared to electroacupuncture and traditional acupuncture, TEAS can avoid discomforts such as pain and bleeding and has higher patient compliance (Chi et al., 2019). In addition, the input time and frequency of the pulse current can be set after setting the parameters, facilitating the quantification and standardization of acupuncture treatment (Xu et al., 2016).

Clinical practice has confirmed that combined rehabilitation training with TEAS can significantly enhance patients’ muscle strength and improve hand function, grip strength, and manual dexterity. This approach facilitates the recovery of upper limb functionality while alleviating pain and addressing symptoms such as muscle spasms, ultimately enhancing the patient’s quality of life (Wang, 2024). In addition, previous network meta-analysis showed that TEAS had the most significant effect on upper limb motor recovery in stroke (Tang et al., 2021). Factors such as the treatment regimen and TEAS parameters should be considered to accurately measure the effectiveness of TEAS. However, no comprehensive meta-analysis has included the above factors simultaneously to specifically analyze the effects of TEAS on upper limb motor recovery in stroke. In this systematic review, stratified analysis was conducted to summarize and analyze the scientific evidence of TEAS on upper limb motor recovery in stroke patients.

## 2 Methods

A systematic review and meta-analysis were conducted according to the reporting checklist of the Preferred Reporting Items for Systematic Reviews and Meta-Analysis (PRISMA) (Page et al., 2021). The study has been registered with Prospero (registration number CRD42024592509). Ethics review board approval was not required for this study.

### 2.1 Search strategy

Two reviewers (LJY and XYW) independently searched databases to collect randomized controlled trials (RCTs) of TEAS on upper limb motor recovery in stroke patients, including eight databases: 4 English databases, Embase, Web of Science, PubMed, The Cochrane Library and 4 Chinese databases: China National Knowledge Infrastructure (CNKI), Wanfang database (WANFANG), VIP database (VIP) and Chinese Biomedical Literature Service system (CBM). The search period covered from the inception of each database to 1st October 2024, and references from the included literature were traced. The languages are Chinese and English. In addition, the key points used in this study include “transcutaneous electrical acupoint stimulation,” “TEAS,” “stroke,” “cerebrovascular accident,” “cerebrovascular disease,” “upper limb motor,” “upper limb,” “randomized controlled trials,” and “clinical trial.” The specific search strategies are described in the Supplementary Appendix.

### 2.2 Eligibility criteria

Studies were eligible for inclusion if they met the following criteria: (1) Study designs: randomized controlled trials; (2) Participants: The selected stroke patients should meet the diagnostic criteria of stroke published in the literature or recognized at home and abroad and be further diagnosed by CT and MRI. The patient had stable vital signs, clear consciousness, and upper limb motor dysfunction. There were no restrictions based on gender, age, or race. (3) Intervention: TEAS stimulation (the acupoints, frequency, duration, and course of TEAS stimulation were not limited); (4) Comparison: sham TEAS treatment or conventional rehabilitation treatment; (5) Outcomes: the primary outcome measure was Fugl Meyer Assessment of the upper extremity (FMA-UE), and the secondary outcome measures were Modified Ashworth Scale (MAS) and Modified Barthel Index (MBI). The FMA-UE is the most commonly used instrument for assessing upper limb motor function in stroke patients and has demonstrated good reliability and validity. Spasticity was measured using the Modified Ashworth Scale (MAS), and the Modified Barthel Index (MBI) was used to evaluate the patient’s ability to perform daily activities.

The exclusion criteria were as follows: (1) conferences and abstract papers; (2) unable to obtain the full text or extract relevant outcome indicators; (3) non-adult patients with stroke; (4) case reports, protocol studies, reviews, and meta-analysis; (5) duplicate published studies; (6) the number of study cases was less than 10; and (7) intervention measures, grouping methods and effect indicators were not consistent.

### 2.3 Study selection and data extraction

Two researchers (WXY and YLJ) independently audited the literature and extracted data. Endnote 20.1 software was used to eliminate duplicate records. Studies that did not meet the criteria were screened by reading titles and abstracts. Finally, the full text was read, the final literature was determined according to the inclusion and exclusion criteria, and the data were extracted. The extracted data were entered into RevMan 5.4 and double-checked for accuracy. If there was any disagreement, the third researcher (WYK) discussed and made a decision. The extracted contents mainly included the name of the first author, the year of publication, the characteristics of participants (age and sample size), the details of TEAS (acupoint selection, frequency, and intervention time), the control intervention, the content of quality assessment (randomized method, blind method, selection report, complete results, and so on), and the data of related outcome indicators.

### 2.4 Risk of bias assessment

Two reviewers (WXY and ZHN) independently used the Cochrane Collaboration tool to assess the risk of bias in the selected trials (Higgins et al., 2011). The evaluation included random sequence generation, allocation concealment, blinding of participants and personnel, blinding of outcome assessment, incomplete outcome data, selective reporting, and other biases. The risk assessment results included high risk, low risk, and unclear. When there were differences in opinions between the two evaluators, they were discussed and resolved first. If the opinions differed, a third evaluator (ZSY) would re-evaluate the results to achieve consistency.

### 2.5 Data synthesis and statistical analysis

Statistical analysis was performed using Review Manager 5.4 and Stata 14.0 software. The outcome indicators involved in this study were all continuous variables. The effect value index was expressed as standard mean difference (SMD), and the 95% confidence interval of each effect value was calculated. If *p* ≥ 0.10, *I^2^* ≤ 50%, there was no heterogeneity or small heterogeneity among the studies, so the fixed effect model was used. If *p* < 0.1, *I^2^* > 50%, the heterogeneity among the studies was considered large, so the random effect model was used (Higgins and Thompson, 2002), and the source for heterogeneity was searched through sensitivity analysis and subgroup analysis. A *p-*value of <0.05 was considered statistically significant. According to the control methods (no TEAS and sham TEAS) and TEAS parameters (including frequency, treatment duration, and retention time) for subgroup analysis. If sufficient trials (≥10 trials) were included, publication bias was assessed using funnel plots and Egger’s test (Egger et al., 1997).

### 2.6 Evidence quality evaluation

The quality of evidence for each outcome was assessed using the Grading of Recommendations Assessment, Development, and Evaluations (GRADE) system. Based on factors such as methodological quality, consistency of results across studies, directness of evidence, precision of evidence, and possibility of publication bias, the evidence level of included RCTs was judged to be downgraded. The quality of evidence was categorized into high, medium, low, or very low (Guyatt et al., 2008). Two researchers conducted the assessments and cross-checked the results. Any disagreement was resolved by consensus. Any disagreements were resolved through consensus, and if consensus could not be reached, a third researcher was consulted.

## 3 Results

### 3.1 Study selection

**Figure 1:**
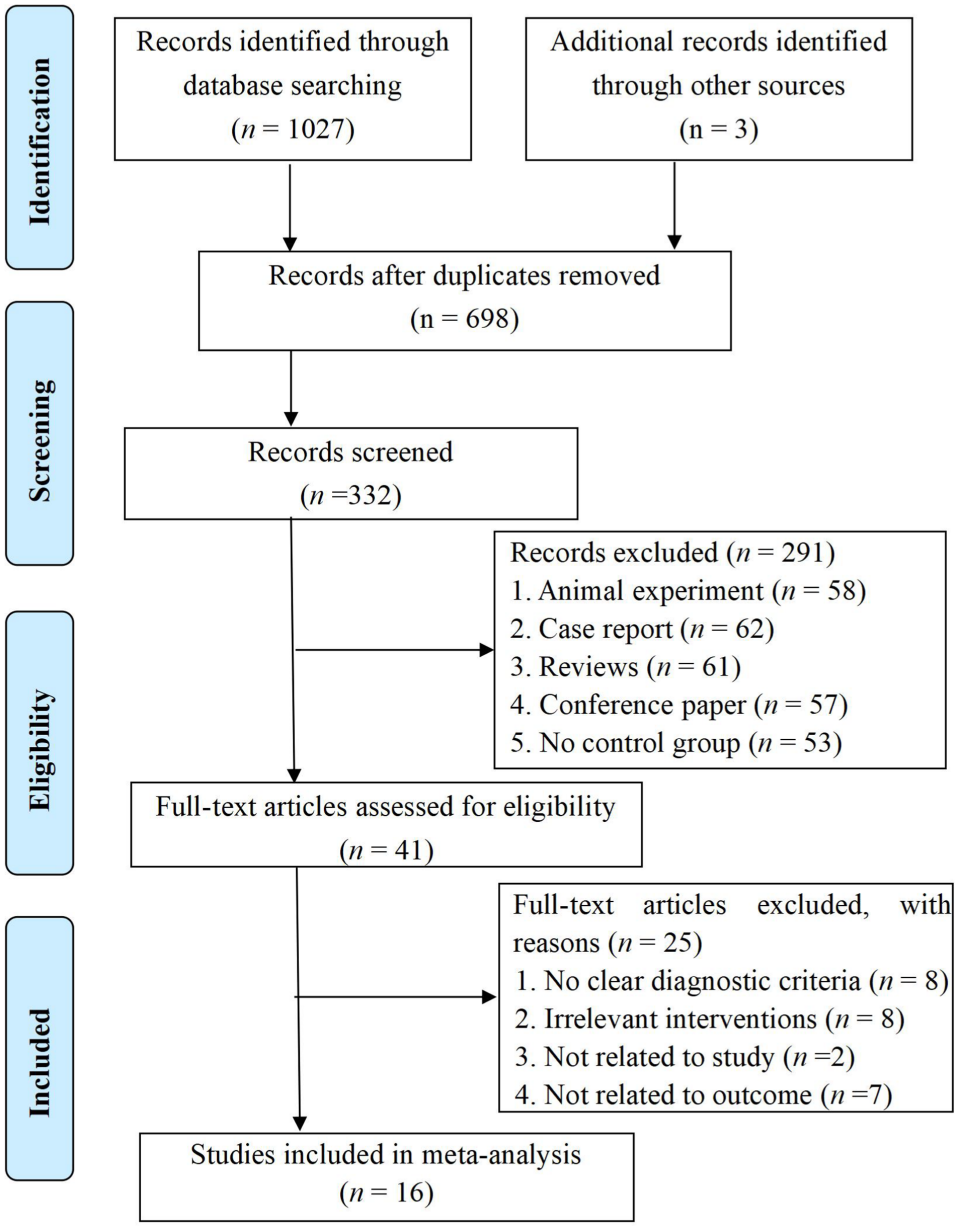
PRISMA flow chart.

We conducted a thorough search of the aforementioned eight databases following our inclusion and exclusion criteria, initially identifying 1,030 articles. After removing duplicates, 332 articles remained in the database. By reviewing the titles and abstracts, we excluded those that did not meet our inclusion criteria, resulting in 41 articles remaining for further consideration. Upon examining the full texts of these articles, we excluded an additional 25 articles. Finally, 16 RCTs (Chao, 2016; Chen et al., 2015; Chen et al., 2019a; Chen et al., 2019b; Gu et al., 2019; Jia et al., 2018; Mao et al., 2017; Peng et al., 2015; Tang et al., 2015; Wang H. et al., 2023; Xia et al., 2021; Xie et al., 2019; Yan et al., 2019; Yang, 2023; Yu et al., 2020; Zheng and Mao, 2020) were included. The flow chart and results of the literature screening are shown in Figure 1.

### 3.2 Study characteristics

The characteristics of the 16 trials included are summarized in Table 1.

**Table 1:** Characteristics of the included studies.

Study	Type of stroke (ICH/CI)	Brunnstrom	Course of disease (days)	Sample size	Age (years)	Gender (M/F)	Intervention		Duration (days)	Outcome
							I	C		
Yang (2023)	I: (19/30) C: (17/32)	/	I: 161.4 ± 15.6 C: 162.6 ± 16.2	98	I: 68.49 ± 2.83 C: 68.51 ± 2.86	I: (25/24) C: (27/22)	TEAS + CR	CR	28	FMA-UE, MAS
Wang D. et al. (2023)	I: (34/68) C: (31/71)	I/V	/	204	I: 60.6 ± 12.5 C: 61.9 ± 10.5	I: (77/25) C: (72/30)	TEAS + CR	Sham TEAS + CR	72	FMA-UE, MBI, MAS
Xia et al. (2021)	I: (15/9) C: (12/12)	II/V	I: 35 ± 18.06 C: 36.4 ± 17.99	48	I: 56.2 ± 9.91 C: 56.5 ± 7.9	I: (17/7) C: (18/6)	TEAS + CR	CR	28	FMA-UE, MBI
Zheng and Mao (2020)	I: (15/19) C: (13/21)	I/V	I: 186 ± 54 C: 177 ± 21	68	I: 58.9 ± 1.3 C: 58.8 ± 1.1	I: (23/11) C: (22/12)	TEAS + CR	CR	28	FMA-UE, MAS
Yu et al. (2020)	I: (24/30) C: (22/32)	/	/	108	I: 62.5 ± 6.5 C: 61.8 ± 5.8	I: (30/24) C: (29/25)	TEAS + CR	CR	30	MBI
Chen et al. (2019a)	I: (5/23) C: (9/19)	< V	I: 152.39 ± 123.46 C: 156.36 ± 123.60	60 (Fall off 4 cases)	I: 64.50 ± 12.95 C: 64.57 ± 9.35	I: (22/6) C: (21/7)	TEAS + CR	CR	42	FMA-UE, MBI
Gu et al. (2019)	I: (6/15) C: (8/13)	/	/	42	I: 63.8 ± 7.1 C: 62.9 ± 8.3	I: (14/7) C: (12/9)	TEAS + CR	CR	28	FMA-UE, MAS
Xie et al. (2019)	I: (CI:42) C: (CI:40)	/	I: 40.28 ± 9.28 C: 41.95 ± 10.88	82	I: 50.38 ± 10.76 C: 49.68 ± 10.52	I: (23/19) C: (24/16)	TEAS + CR	CR	28	FMA-UE, MBI
Yan et al. (2019)	/	/	/	120	I: 62.40 C: 60.90	I: (33/27) C: (39/21)	TEAS + CR	CR	120	MBI
Chen et al. (2019b)	I: (8/18) C: (6/21)	III/V	I: 275.95 ± 59.11 C: 271.43 ± 74.01	53	I: 60.75 ± 16.62 C: 60.45 ± 11.25	I: (19/7) C: (20/7)	TEAS + CR	CR	42	FMA-UE, MBI, MAS
Jia et al. (2018)	I: (22/32) C: (20/34)	I/V	I: 144 ± 51 C: 150 ± 48	108	I: 64.7 ± 9.8 C: 64.2 ± 9.5	I: (29/25) C: (32/22)	TEAS + CR	CR	84	MBI
Mao et al. (2017)	I: (12/14) C: (11/15)	I/V	I: 177.60 ± 72.00 C: 186.90 ± 96.30	52	I: 59.52 ± 7.35 C: 58.92 ± 6.21	I: (14/12) C: (18/8)	TEAS + CR	CR	28	FMA-UE, MAS
Chao (2016)	I: (17/10) C: (12/16)	/	I: 161.4 ± 104.1 C: 169.2 ± 101.7	55	I: 52.26 ± 15.56 C: 49.54 ± 11.21	I: (22/5) C: (24/4)	TEAS + CR	Sham TEAS + CR	28	FMA-UE, MBI
Peng et al. (2015)	I: (7/14) C: (7/13)	< V	I: 67.5 ± 26.1 C: 60.6 ± 31.5	46 (Fall off 5 cases)	I: 66.4 ± 10.8 C: 65.5 ± 11.2	I: (9/12) C: (10/10)	TEAS + CR	Sham TEAS + CR	21	FMA-UE, MBI
Chen et al. (2015)	I: (7/13) C: (8/15)	I/V	I: 186.00 ± 85.50 C: 185.10 ± 92.40	43	I: 61.05 ± 9.24 C: 57.69 ± 8.79	I: (17/3) C: (19/4)	TEAS + CR	CR	28	FMA-UE, MBI, MAS
Tang et al. (2015)	I: (4/11) C: (6/10)	I/II	I: 40.23 ± 16.02 C: 37.64 ± 18.32	31	I: 54.69 ± 9.68 C: 57.93 ± 11.2	I: (12/3) C: (14/2)	TEAS + CR	CR	42	FMA-UE, MBI

I, Intervention group; C, Control group; ICH, Intra Cerebral Hemorrhage; CI, Cerebral Infarction; M, Male; F, Famale; /, not mention; CR, Conventional Rehabilitation; TEAS, Transcutaneous electrical acupoint stimulation; FMA-UE, Fugl-Meyer Assessment of the upper extremity; MAS, Modified Ashworth Scale; MBI, Modified Barthel Index.

A total of 16 trials involving 1,218 stroke patients (10 dropouts) were included in this meta-analysis, with 603 in the TEAS group and 605 in the control group. The trials were published between 2015 and 2023. Sample sizes ranged from 31 to 204. The sample included 431 women and 777 men. As described in the included trials, except for one trial (Yan et al., 2019), which did not report the type of stroke, the remaining trials included 377 patients with intra-cerebral hemorrhage and 712 patients with cerebral infarction. A total of 10 trials (Chen et al., 2015; Chen et al., 2019a; Chen et al., 2019b; Jia et al., 2018; Mao et al., 2017; Peng et al., 2015; Tang et al., 2015; Wang H. et al., 2023; Xia et al., 2021; Zheng and Mao, 2020) specified Brunstrom stages. Moreover, three trials (Chen et al., 2015; Peng et al., 2015; Wang H. et al., 2023) used sham TEAS as the control group. The duration of treatment ranged from 21 to 120 days. The outcome measures included the FMA-UE, MAS, and MBI. A total of 13 trials (Chao, 2016; Chen et al., 2015; Chen et al., 2019b; Mao et al., 2017; Peng et al., 2015; Tang et al., 2015; Wang H. et al., 2023; Xia et al., 2021; Yang, 2023; Zheng and Mao, 2020) used FMA-UE as an outcome measure, seven trials (Chen et al., 2015; Chen et al., 2019b; Gu et al., 2019; Mao et al., 2017; Wang H. et al., 2023; Yang, 2023; Zheng and Mao, 2020) used MAS, and 11 trials (Chen et al., 2015; Chen et al., 2019a; Chen et al., 2019b; Jia et al., 2018; Peng et al., 2015; Tang et al., 2015; Wang H. et al., 2023; Xia et al., 2021; Xie et al., 2019; Yan et al., 2019; Yu et al., 2020) used MBI.

### 3.3 TEAS regimen

The details of TEAS for the included 16 trials are summarized in Table 2. For stimulation frequencies, 100 Hz and 2 Hz were popular across trials. The most commonly used acupoints were LI10 (Shousanli) and TE5 (Waiguan), used in 13 trials. The most commonly used treatment duration was 30 min (78.5%). The most commonly used frequency of treatment was 5 times per week (66.7%).

**Table 2:** Details of transcutaneous electrical acupoint stimulation in included trials.

Study	Frequency (Hz)/intensity (mA) of electrical stimulation	The site of electrical stimulation	Duration of treatments	Number of treatments	Frequency (weeks/days)
Yang (2023)	35 Hz/10/50 mA	LI10 (Shousanli), TE5 (Waiguan)	15 min	TEAS + CR: 28 CR: 28	Once a day
Wang D. et al. (2023)	2 Hz	LI10 (Shousanli), TE5 (Waiguan)	30 min	TEAS + CR: 30 Sham TEAS + CR: 30	5 times per week
Xia et al. (2021)	100 Hz/100 mA	LI15 (Jianyu), LI11 (Quchi), LI10 (Shousanli), TE5 (Waiguan)	20 min	TEAS + CR: 24 CR: 24	6 times per week
Zheng and Mao (2020)	/	LI10 (Shousanli), TE5 (Waiguan)	30 min	TEAS + CR: 34 CR: 34	5 times per week
Yu et al. (2020)	100 Hz/10/40 mA	Eight evil points	30 min	TEAS + CR: 54 CR: 54	Once a day
Chen et al. (2019a)	Brunnstrom I ~ II: 2 Hz, Brunnstrom III ~ IV: 4/15 Hz	LI10 (Shousanli), TE5 (Waiguan)	30 min	TEAS + CR: 28 CR: 28	5 times per week
Gu et al. (2019)	35 Hz/10/50 mA	LI10 (Shousanli), TE5 (Waiguan)	15 min	TEAS + CR: 21 CR: 21	Once a day
Xie et al. (2019)	2 Hz	TE5 (Waiguan), LI15 (Jianyu), LI11 (Quchi), LI10 (Shousanli)	30 min	TEAS + CR: 42 CR: 40	5 times per week
Yan et al. (2019)	General condition: 10 Hz, The disease is serious: 4 Hz	LI10 (Shousanli), TE5 (Waiguan)	30 min	CT + TEAS: 60 CT: 60	/
Chen et al. (2019b)	Brunnstrom III ~ IV: 10/15 Hz Brunnstrom V: 4 Hz	LI10 (Shousanli), TE5 (Waiguan)	30 min	TEAS + CR: 26 CR: 27	5 times per week
Jia et al. (2018)	Brunnstrom I/II: 2 Hz, Brunnstrom III/IV: 4/15 Hz, Brunnstrom V/VI: 4 Hz	LI10 (Shousanli), TE5 (Waiguan)	Brunnstrom I/II: 25/30 min, Brunnstrom III/IV: 20/30 min, Brunnstrom V/VI: 30 min	TEAS + CR: 54 CR: 54	5 times per week
Mao et al. (2017)	/	LI10 (Shousanli), TE5 (Waiguan)	30 min	TEAS + CR: 26 CR: 26	5 times per week
Chao (2016)	100 Hz/10/40 mA	Eight evil points	30 min	TEAS + CR: 28 Sham TEAS + CR: 28	Once a day
Peng et al. (2015)	100 Hz	TE5 (Waiguan), LI15 (Jianyu), LI11 (Quchi), LI14 (Hegu)	30 min	TEAS + CR: 21 Sham TEAS + CR: 20	5 times per week
Chen et al. (2015)	/	LI10 (Shousanli), TE5 (Waiguan)	/	TEAS + CR: 20 CR: 23	5 times per week
Tang et al. (2015)	/	LI10 (Shousanli), TE5 (Waiguan)	30 min	TEAS + CR: 30 CR: 30	5 times per week

CR, Conventional Rehabilitation; TEAS, Transcutaneous electrical acupoint stimulation; /, not mention.

### 3.4 Risk of bias

**Figure 2:**
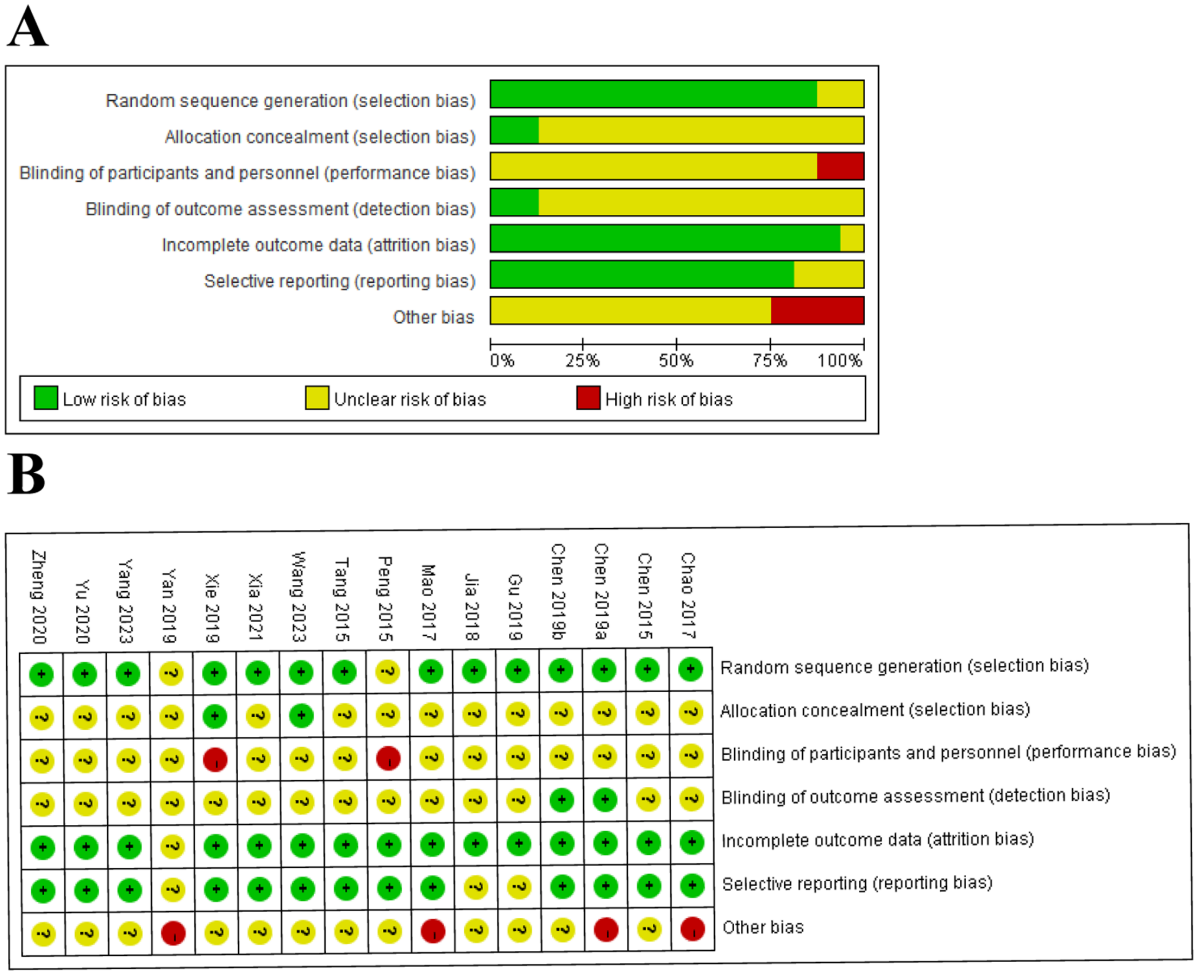
Risk of bias (ROB) assessments of included studies. **(A)** ROB graph. **(B)** ROB summary.

A total of 14 (Chao, 2016; Chen et al., 2015; Chen et al., 2019a; Chen et al., 2019b; Gu et al., 2019; Jia et al., 2018; Mao et al., 2017; Tang et al., 2015; Wang H. et al., 2023; Xia et al., 2021; Xie et al., 2019; Yang, 2023; Yu et al., 2020; Zheng and Mao, 2020) trials described detailed methods of randomization, except for two trials (Peng et al., 2015; Yan et al., 2019) in which the risks were not clear. Only two trials (Wang H. et al., 2023; Xie et al., 2019) emphasized allocation concealment. Two trials (Peng et al., 2015; Xie et al., 2019) performed a blinding method on subjects and operators as high-risk, and only two trials (Chen et al., 2019a; Chen et al., 2019b) performed blinding to outcome assessment. One trial (Yan et al., 2019) was conducted as an unclear risk due to incomplete outcome data. In addition, three trials (Gu et al., 2019; Jia et al., 2018; Yan et al., 2019) had unclear selective reporting bias, and four trials (Chao, 2016; Chen et al., 2019a; Mao et al., 2017; Yan et al., 2019) reported other biases. The risk of bias is summarized in Figure 2.

### 3.5 Meta-analysis

#### 3.5.1 Fugl Meyer Assessment of the Upper Extremity (FMA-UE)

**Figure 3:**
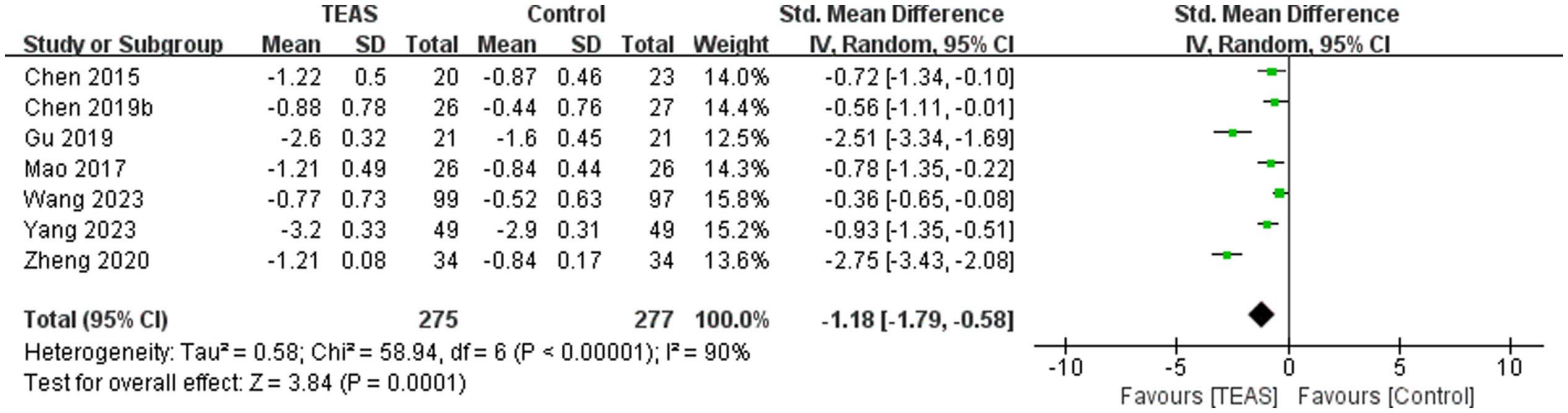
Forest plot and meta-analysis of FMA-UE.

**Figure 4:**
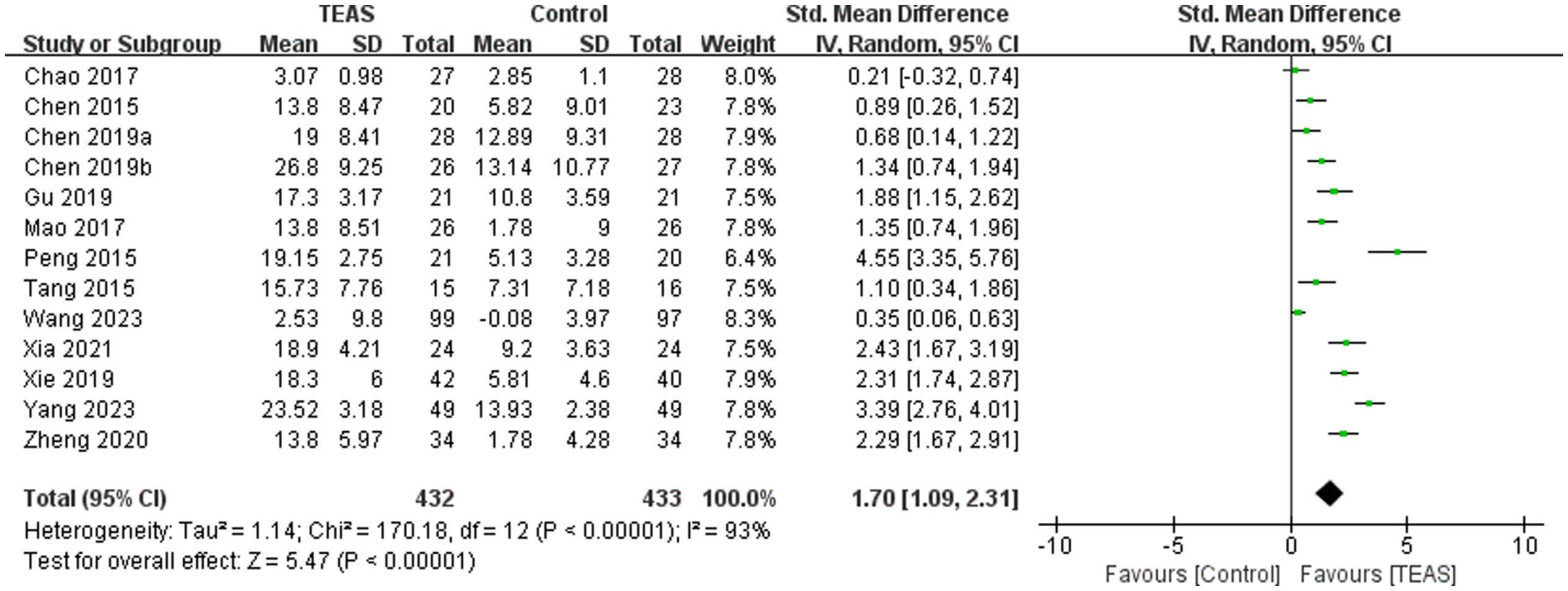
Subgroup analysis of FMA-UE is based on the types of control groups.

A total of 13 RCTs (Chao, 2016; Chen et al., 2015; Chen et al., 2019b; Mao et al., 2017; Peng et al., 2015; Tang et al., 2015; Wang H. et al., 2023; Xia et al., 2021; Yang, 2023; Zheng and Mao, 2020) involving 865 patients reported FMA-UE. There was a significant difference in FMA-UE in the TEAS group (SMD = 1.70, 95CI% = 1.09 to 2.31, *p* < 0.00001, *I^2^* = 93%; Figure 3), although the quality of evidence was low (Table 3). The results of subgroup analysis showed that TEAS had a statistically significant effect on improving FMA-UE compared with no TEAS (Figure 4) (SMD = 1.76, 95CI% = 1.22 to 2.30, *p* < 0.00001, *I^2^* = 86%; Table 4), while there was no significant statistical difference compared with sham TEAS (SMD = 1.54, 95CI% = 0.00 to 3.09, *p* = 0.05, *I^2^* = 96%; Table 4).

**Table 3:** GRADE of evidence of outcomes of the included trials.

Outcomes	Certainty assessment						No. of patients	Effect sizes	Certainty
	No. of trials	Risk of bias	Inconsistency	Indirectness	Imprecision	Publication bias	Intervention/Control	SMD (95% CI)	
FMA-UE	13	Serious	Serious	No serious	No serious	Undetected	432/433	SMD 1.70 (1.09,2.31)	⊕⊕⊝⊝ Low
MAS	7	Serious	Serious	No serious	No serious	Serious	275/277	SMD -1.18 (−1.79,-0.58)	⊕⊝⊝⊝ Very Low
MBI	11	Serious	Serious	No serious	No serious	Undetected	443/443	SMD 1.53 (0.85,2.20)	⊕⊕⊝⊝ Low

SMD, standard mean difference; CI, confidence interval; GRADE, Grading of Recommendations Assessment, Development, and Evaluation.

Risk of bias: serious, study with unclear risk of bias; Inconsistency: Serious, *I*^2^ > 50%.

Indirectness: no indirectness of evidence was found in any study.

Imprecision (based on sample size): Serious, *n* < 500 participants.

Publication bias: Undetected due to the number of trials less than the recommended arbitrary minimum number of 10.

**Table 4:** The results of subgroup analyses.

			No. of studies	No. of patients	SMD (95% CI)	*p*	*I^2^*	Effect model
FMA-UE	Types of control group	TEAS + CR vs. CR	10	573	1.76 (1.22, 2.30)	<0.00001	86%	Random
		TEAS + CR vs. Sham TEAS + CR	3	292	1.54 (0.00, 3.09)	0.05	96%	Random
	Frequency of TEAS	Once a day	3	195	1.82 (−0.13, 3.77)	0.07	97%	Random
		5 times per week	9	622	1.57 (0.92, 2.22)	<0.00001	92%	Random
		6 times per week	1	48	2.43 (1.67, 3.19)	<0.00001	/	/
	Treatment duration	0–4 weeks	9	529	2.09 (1.33, 2.86)	<0.00001	92%	Random
		5–8 weeks	3	140	1.01 (0.60, 1.42)	<0.00001	24%	Random
		>8 weeks	1	196	0.35 (0.06, 0.63)	0.02	/	/
	TEAS retention time	<30 min	3	188	2.58 (1.68, 3.49)	<0.00001	80%	Random
		≥30 min	9	600	1.50 (0.83, 2.16)	<0.0001	92%	Random
MAS	Types of control group	TEAS + CR vs. CR	6	356	−1.34 (−2.03, −0.65)	0.0001	88%	Random
		TEAS + CR vs. Sham TEAS + CR	1	196	−0.36 (−0.65, −0.08)	0.01	/	/
	Frequency of TEAS	Once a day	2	140	−1.68 (−3.23, −0.13)	0.03	91%	Random
		5 times per week	5	412	−1.01 (−1.74, −0.28)	0.007	90%	Random
	Treatment duration	0–4 weeks	5	303	−1.51 (−2.30, −0.71)	0.0002	89%	Random
		5–8 weeks	1	53	−0.56 (−1.11, −0.01)	0.04	/	/
		>8 weeks	1	196	−0.36 (−0.65, −0.08)	0.01	/	/
	TEAS retention time	<30 min	2	140	−1.68 (−3.23, −0.13)	0.03	91%	Random
		≥30 min	4	369	−1.09 (−2.00, 0.17)	0.02	93%	Random
MBI	Types of control group	TEAS + CR vs. CR	9	649	1.52 (0.84, 2.21)	<0.0001	93%	Random
		TEAS + CR vs. Sham TEAS + CR	2	237	1.61 (−1.59, 4.81)	0.32	98%	Random
	Frequency of TEAS	Once a day	1	108	0.51 (0.13, 0.90)	0.009	/	/
		5 times per week	8	610	1.48 (0.58, 2.37)	0.001	95%	Random
		6 times per week	1	48	3.67 (2.72, 4.62)	<0.00001	/	/
	Treatment duration	0–4 weeks	3	173	2.53 (0.54, 4.52)	0.01	95%	Random
		5–8 weeks	5	289	1.17 (0.39, 1.94)	0.003	88%	Random
		>8 weeks	3	299	2.18 (1.03, 3.32)	0.0002	92%	Random
	TEAS retention time	<30 min	1	48	3.67 (2.72, 4.62)	<0.00001	/	/
		≥30 min	8	687	1.28 (0.57, 2.00)	0.0004	94%	Random

CR, Conventional Rehabilitation; TEAS, Transcutaneous electrical acupoint stimulation; /, not mention; FMA-UE, Fugl-Meyer Assessment of the upper extremity; MAS, Modified Ashworth Scale; MBI, Modified Barthel Index.

**Figure 5:**
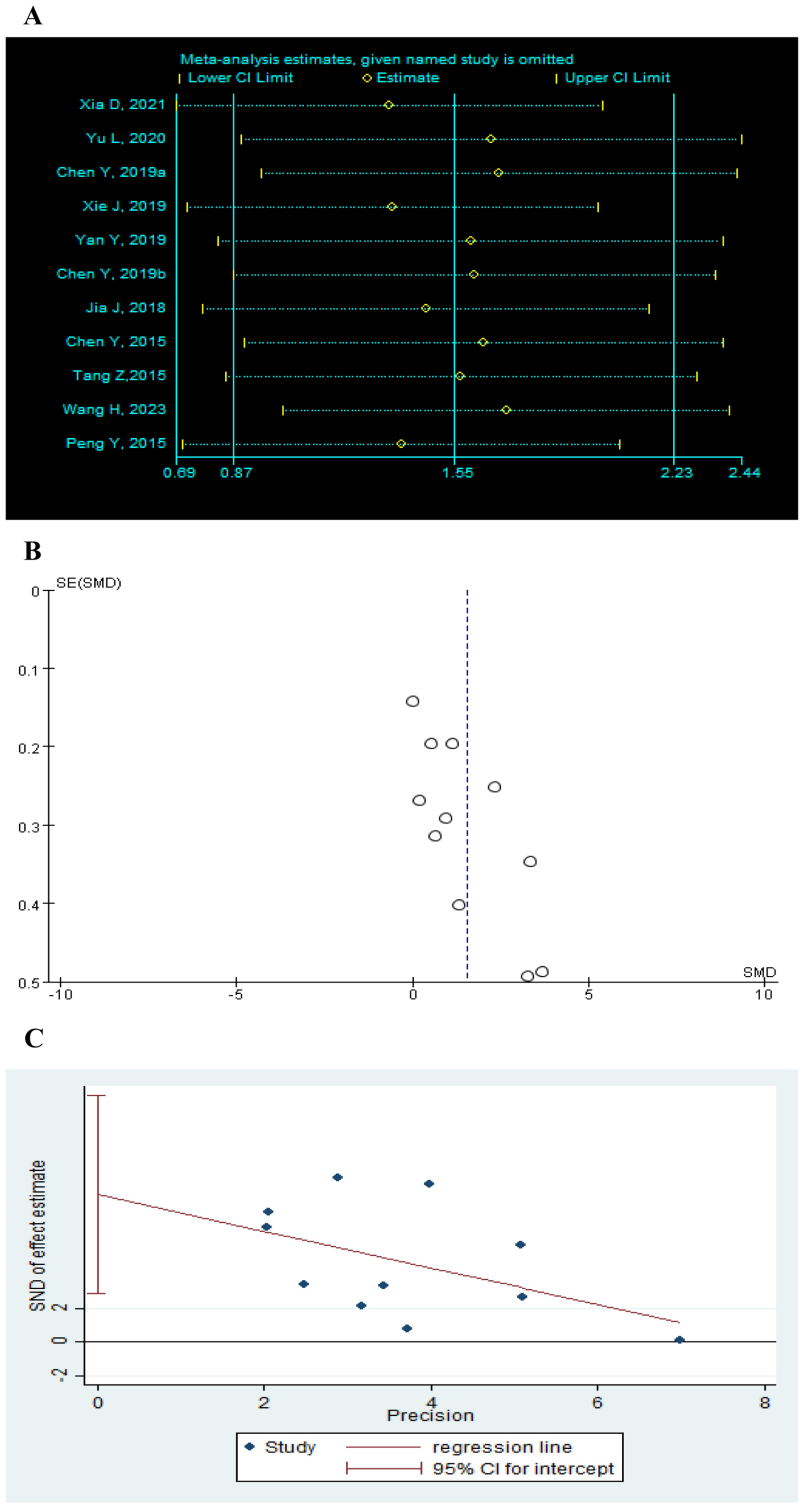
Subgroup analysis of FMA-UE based on TEAS parameters: **(A)** Frequency of TEAS. **(B)** Treatment duration. **(C)** TEAS retention time.

In addition, we also performed the subgroup analysis of the TEAS parameters (Figure 5). In the subgroup analysis of frequency of TEAS (Figure 5A), there was a statistically significant difference between the 5 times per week, and 6 times per week TEAS treatment (SMD = 1.57, 95CI% = 0.92 to 2.22, *p* < 0.00001, *I^2^* = 92%; SMD = 2.43, 95CI% = 1.67 to 3.19, *p* < 0.00001; Table 4), while there was no significant difference between TEAS treatment once a day (SMD = 1.82, 95CI% = −0.13 to 3.77, *p* = 0.07, *I^2^* = 97%; Table 4). Subgroup based on the treatment duration (Figure 5B): 0–4 weeks, 5–8 weeks, and more than 8 weeks, TEAS was superior to the control group in improving FMA-UE (SMD = 2.09, 95CI% = 1.33 to 2.86, *p* < 0.00001, *I^2^* = 92%; SMD = 1.01, 95CI% = 0.60 to 1.42, *p* < 0.00001; SMD = 0.35, 95CI% = 0.06 to 0.63, *p* = 0.02; Table 4). Furthermore, in the subgroup analysis of retention time (Figure 5C), TEAS was superior to the control group in improving FMA-UE either less than 30 min or more than 30 min (SMD = 2.58, 95CI% = 1.68 to 3.49, *p* < 0.00001, *I^2^* = 80%; SMD = 1.50, 95CI% = 0.83 to 2.16, *p* < 0.0001, *I^2^* = 92%; Table 4).

#### 3.5.2 Modified Ashworth Scale (MAS)

**Figure 6:**
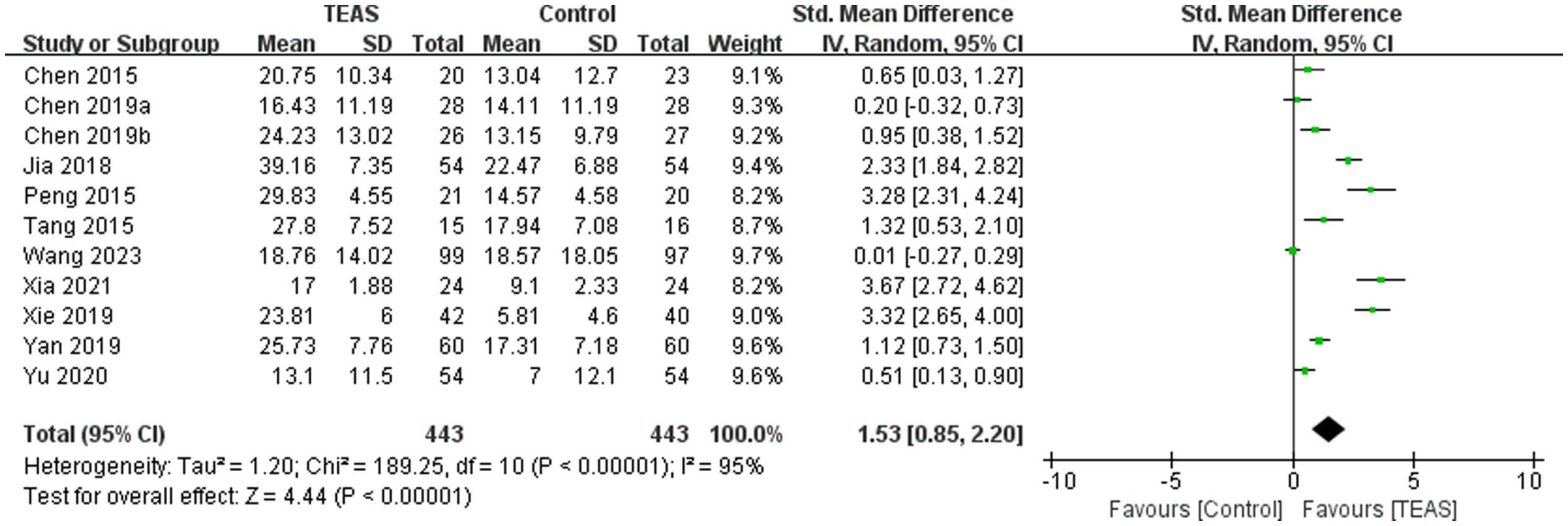
Forest plot and meta-analysis of MAS.

**Figure 7:**
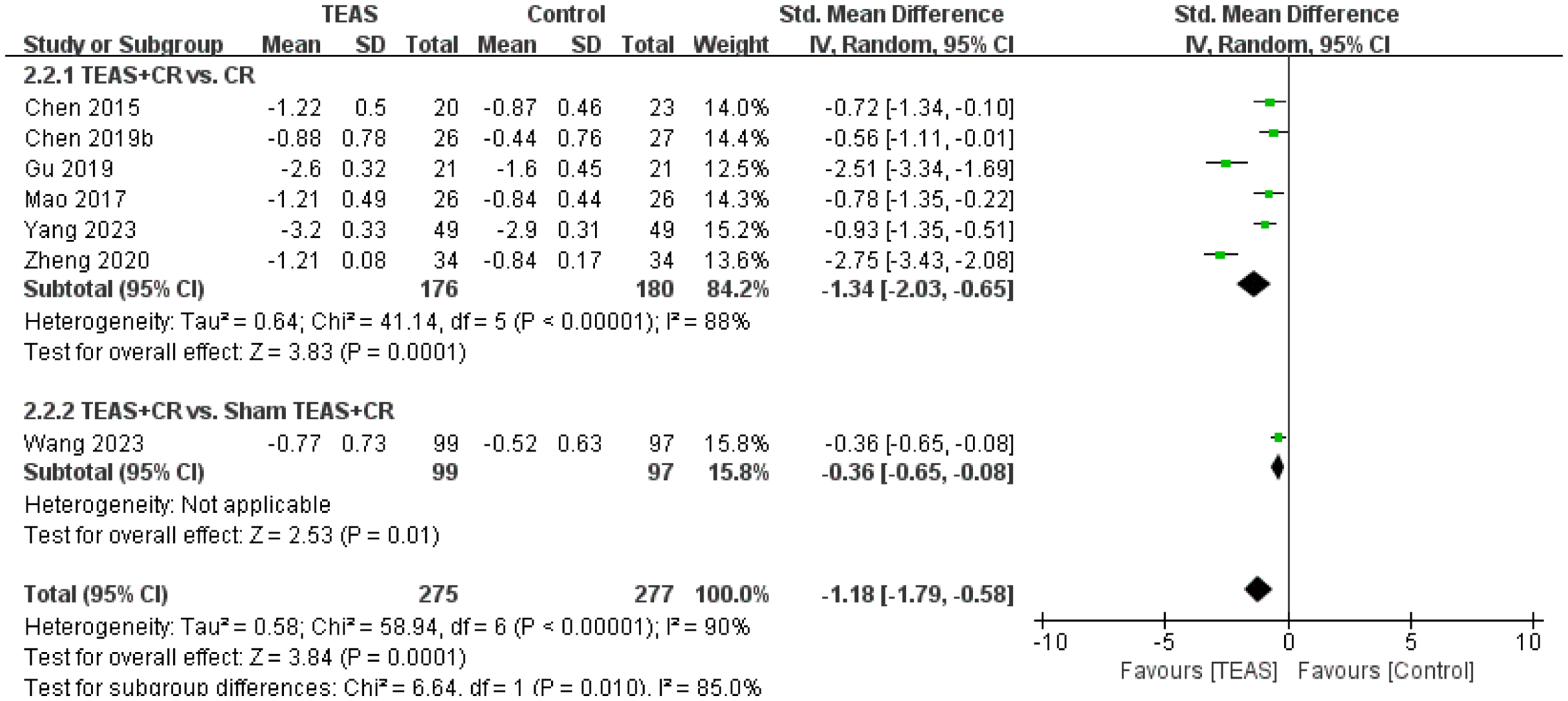
Subgroup analysis of MAS is based on the types of control groups.

A total of 7 RCTs (Chen et al., 2015; Chen et al., 2019b; Gu et al., 2019; Mao et al., 2017; Wang H. et al., 2023; Yang, 2023; Zheng and Mao, 2020) reported MAS in a total of 552 patients. Meta-analysis results show that compared with the control group, the TEAS group had significant improvement in MAS (SMD = −1.18, 95CI% = −1.79 to −0.58, *p* < 0.00001, *I^2^* = 90%; Figure 6), although the quality of evidence was very low (Table 3). The results of subgroup analysis showed that TEAS had significant advantages in improving MAS compared with no TEAS and sham TEAS (Figure 7) (SMD = −1.34, 95CI% = −2.03 to −0.65, *p* = 0.0001, *I^2^* = 88%; SMD = −0.36, 95CI% = −0.65 to −0.08, *p* = 0.01; Table 4).

**Figure 8:**
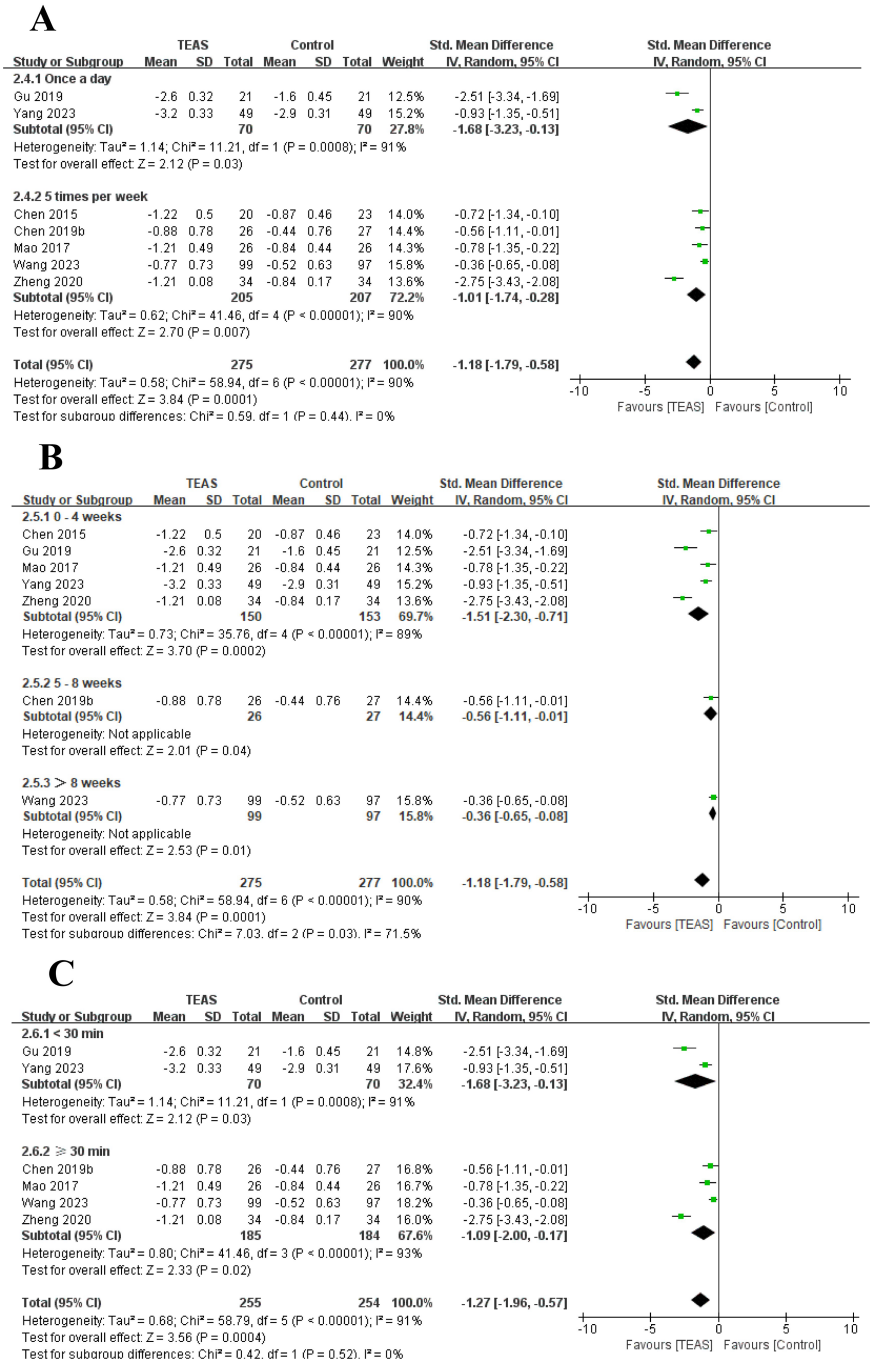
Subgroup analysis of MAS based on TEAS parameters: **(A)** Frequency of TEAS. **(B)** Treatment duration. **(C)** TEAS retention time.

We performed the subgroup analysis of the TEAS parameters (Figure 8). In the subgroup analysis of the frequency of TEAS (Figure 8A), once a day and 5 times per week had statistical significance in the evaluation of MAS (SMD = −1.68, 95CI% = −3.23 to −0.13, *p* = 0.03, *I^2^* = 91%; SMD = −1.01, 95CI% = −1.74 to −0.28, *p* = 0.007; Table 4). Subgroup analysis based on the treatment duration (Figure 8B): 0–4 weeks, 5–8 weeks, and more than 8 weeks, TEAS was superior to the control group in improving MAS (SMD = −1.51, 95CI% = −2.30 to −0.71, *p* = 0.0002, *I^2^* = 89%; SMD = −0.56, 95CI% = −1.11 to −0.01, *p* = 0.04; SMD = −0.36, 95CI% = −0.65 to −0.08, *p* = 0.01; Table 4). Furthermore, in the subgroup analysis of retention time (Figure 8C), TEAS for less than 30 min or more than 30 min was superior to the control group in improving MAS (SMD = −1.68, 95CI% = −3.23 to −0.13, *p* = 0.03, *I^2^* = 91%; SMD = 1.09, 95CI% = 2.00 to 0.17, *p* = 0.02, *I^2^* = 93%; Table 4).

#### 3.5.3 Modified Barthel Index (MBI)

**Figure 9:**
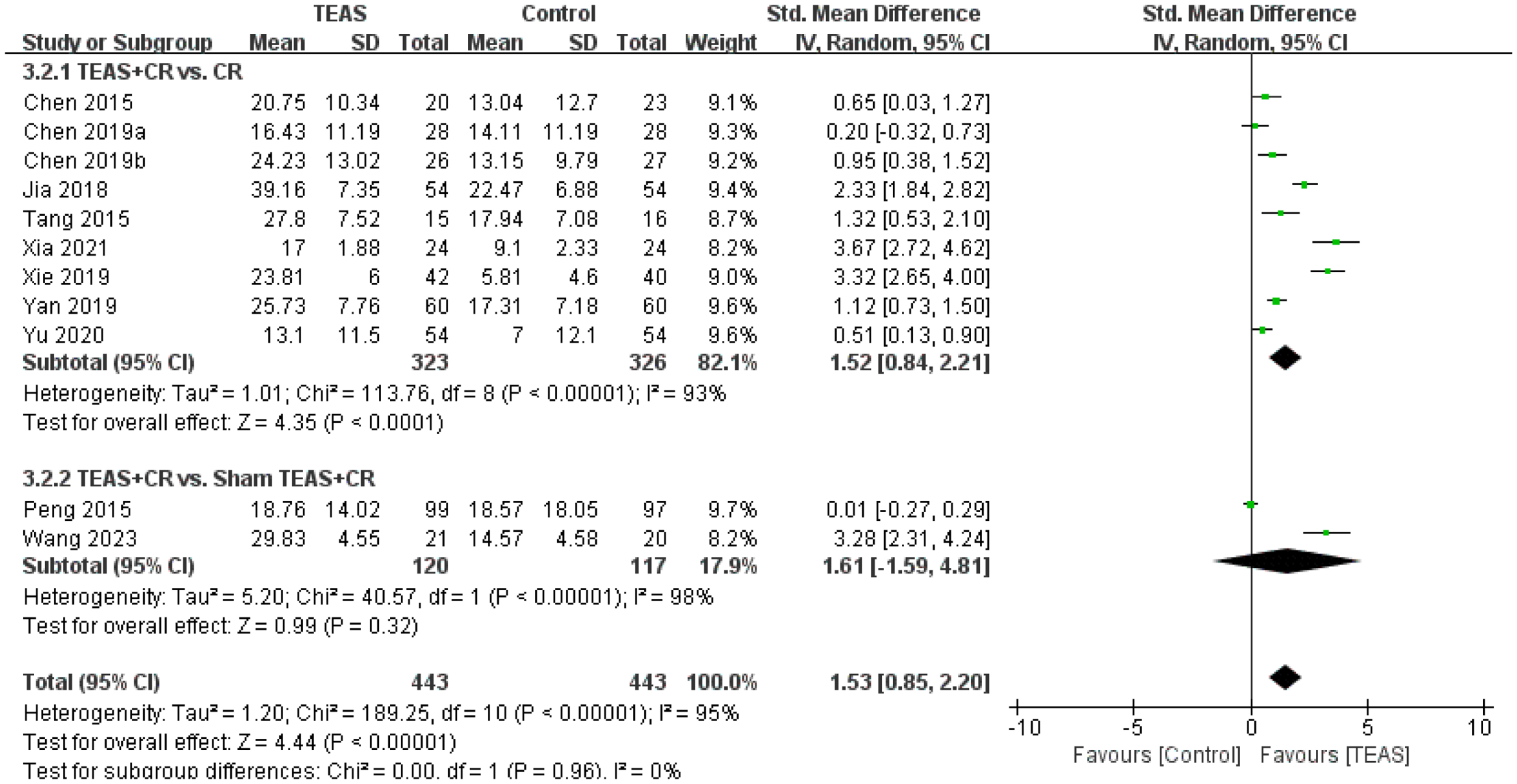
Forest plot and meta-analysis of MBI.

A total of 11 RCTs (Chen et al., 2015; Chen et al., 2019a; Chen et al., 2019b; Jia et al., 2018; Peng et al., 2015; Tang et al., 2015; Wang H. et al., 2023; Xia et al., 2021; Xie et al., 2019; Yan et al., 2019; Yu et al., 2020) involving 886 patients reported MBI. Meta-analysis results showed that compared with the control group, the TEAS group had significant differences in MBI (SMD = 1.53, 95CI% = 0.85 to 2.20, *p* < 0.00001, *I^2^* = 95%; Figure 9), although the quality of evidence was low (Table 3).

**Figure 10:**
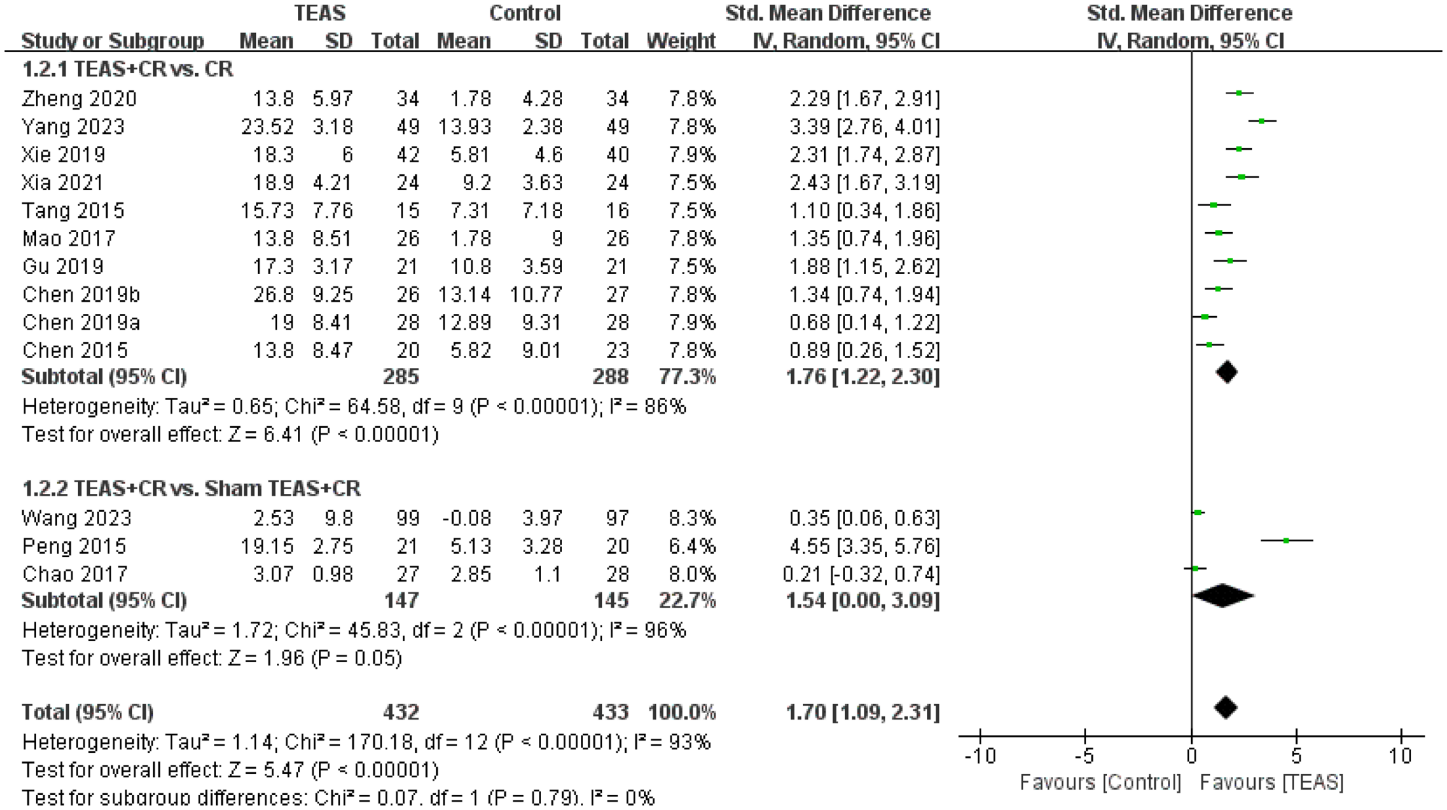
Subgroup analysis of MBI is based on the types of control groups.

Subgroup analysis results show that, compared with no TEAS (Figure 10), TEAS was statistically different in improving the MBI. (SMD = 1.52, 95CI % = 0.84 to 2.21, *p* < 0.0001, *I^2^* = 93%; Table 4), although there was no significant statistical difference compared with sham TEAS (SMD = 1.61, 95CI% = −1.59 to 4.81, *p* = 0.32, *I^2^* = 98%; Table 4).

**Figure 11:**
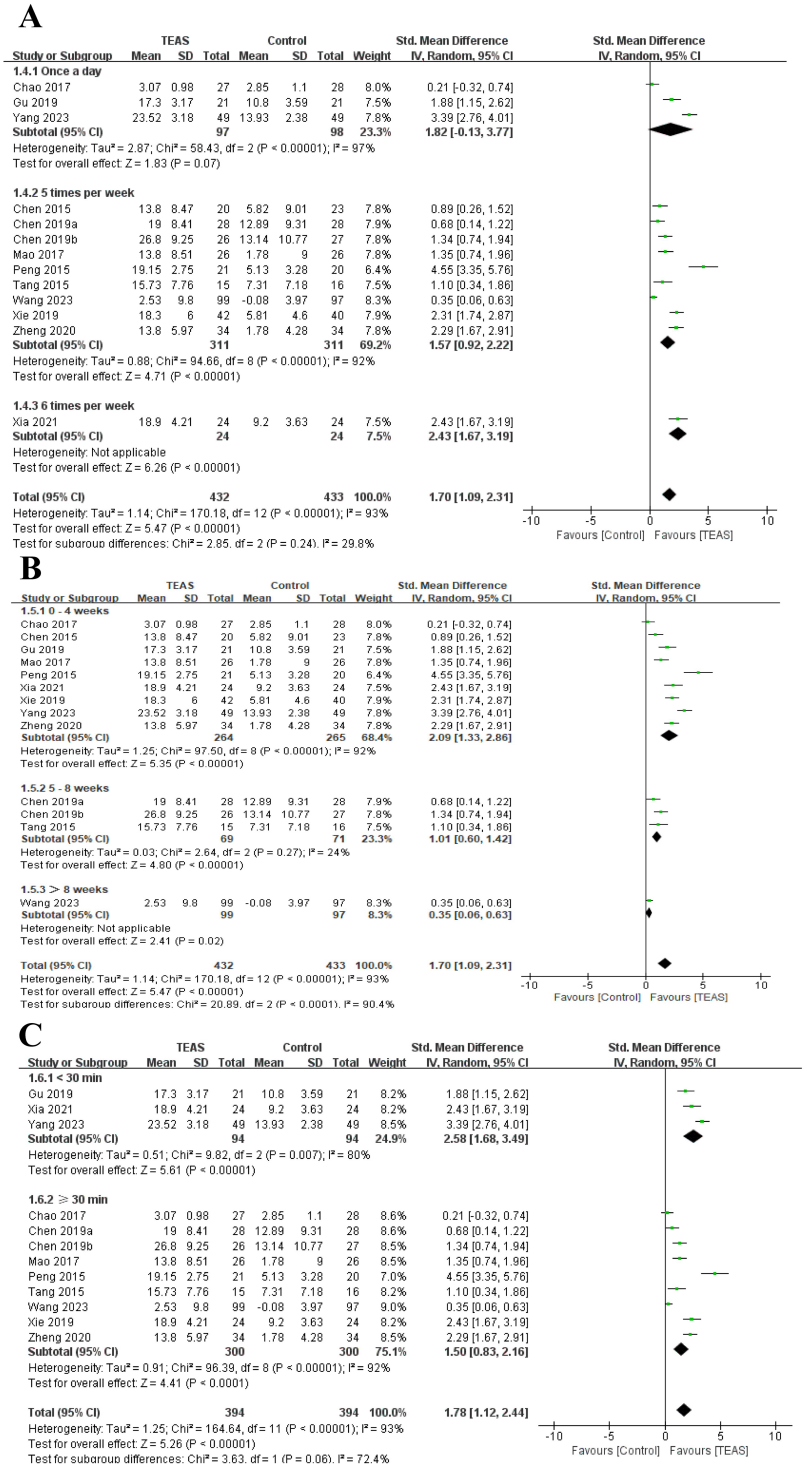
Subgroup analysis of MBI based on TEAS parameters: **(A)** Frequency of TEAS. **(B)** Treatment duration. **(C)** TEAS retention time.

In addition, we performed a subgroup analysis of the TEAS parameters (Figure 11). In the subgroup analysis of frequency of TEAS (Figure 11A), TEAS treatment administered once a day, 5 times per week, and 6 times per week showed statistical significance in the evaluation of MBI (SMD = 0.51, 95CI% = 0.13 to 0.90, *p* = 0.009; SMD = 1.48, 95CI% = 0.58 to 2.37, *p* = 0.001, *I^2^* = 95%; SMD = 3.67, 95CI% = 2.72 to 4.62, *p* < 0.00001; Table 4). Subgroup based on the treatment duration (Figure 11B): 0–4 weeks, 5–8 weeks, and more than 8 weeks, TEAS was superior to the control group in improving MBI (SMD = 2.53, 95CI% = 0.54 to 4.52, *p* = 0.01, *I^2^* = 95%; SMD = 1.17, 95CI% = 0.39 to 1.94, *p* = 0.003, *I^2^* = 88%; SMD = 2.18, 95CI% = 1.03 to 3.32, *p* = 0.0002, *I^2^* = 92%; Table 4). Furthermore, in the subgroup analysis of retention time (Figure 11C), TEAS was superior to the control group in improving MBI either less than 30 min or more than 30 min (SMD = 3.67, 95CI% = 2.72 to 4.62, *p* < 0.00001; SMD = 1.28, 95CI% = 0.57 to 2.00, *p* = 0.0004, *I^2^* = 94%; Table 4).

#### 3.5.4 Safety of intervention

Only one trial (Wang H. et al., 2023) mentioned the safety of TEAS intervention, and no adverse reactions were reported. All the included trials reported that patients could tolerate TEAS intervention well without obvious adverse reactions. Therefore, larger-scale RCTs are needed to further confirm and report the safety of TEAS in the treatment of upper limb dysfunction after stroke.

#### 3.5.5 Sensitivity analysis and publication bias

**Figure 12:**
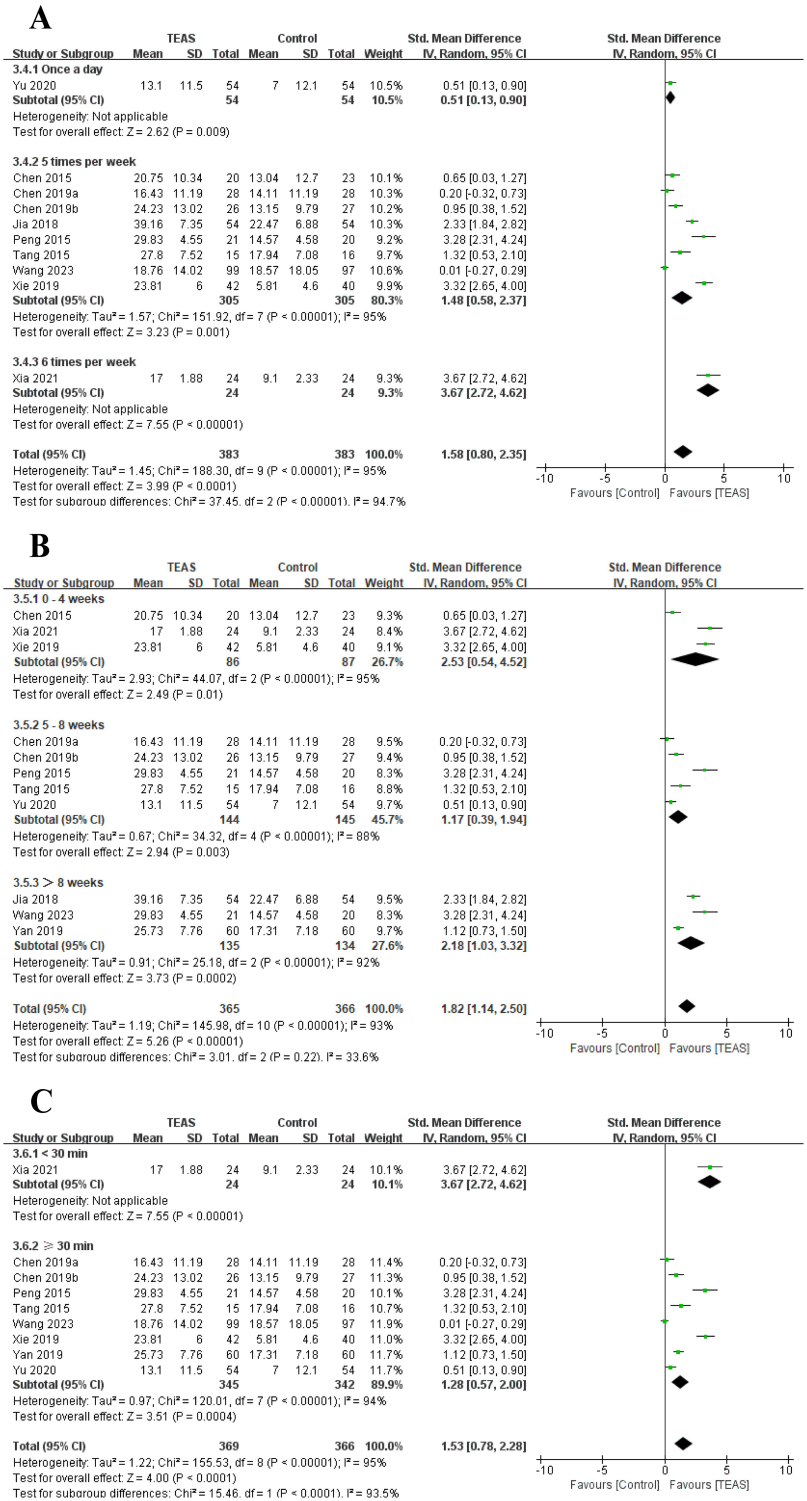
Effect of TEAS on FMA-UE. **(A)** Sensitivity analysis of FMA-UE. **(B)** A funnel plot analysis revealed potential publication bias. **(C)** Egger’s test included studies on FMA-UE.

**Figure 13:**
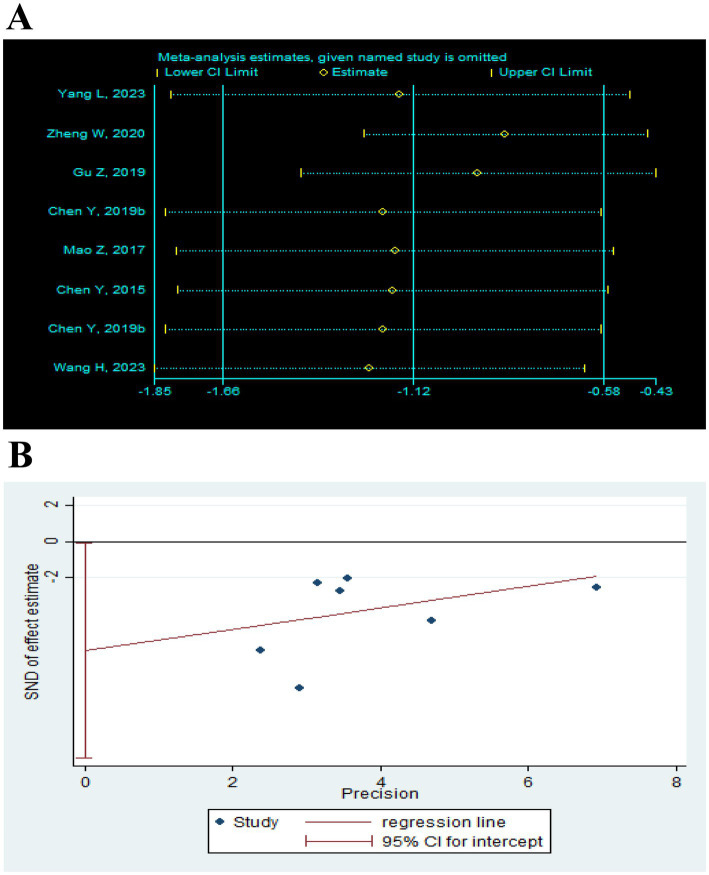
Effect of TEAS on MAS. **(A)** Sensitivity analysis of MAS. **(B)** Egger’s test included studies on MAS.

**Figure 14:**
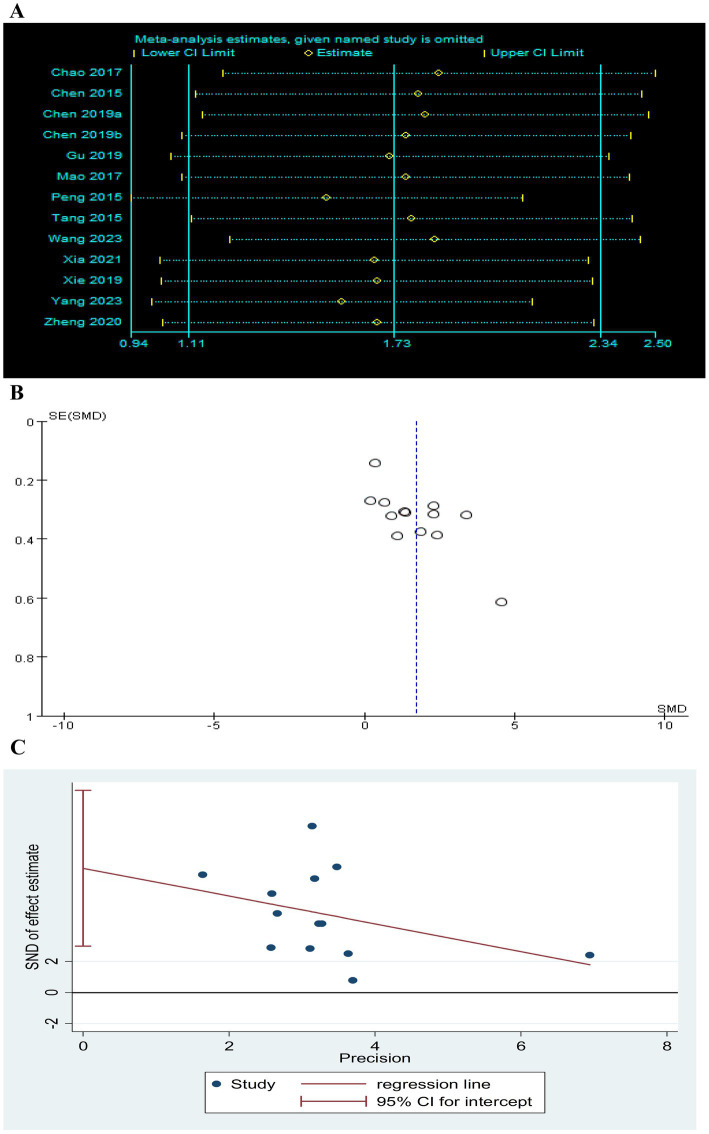
Effect of TEAS on MBI. **(A)** Sensitivity analysis of MBI. **(B)** A funnel plot analysis revealed potential publication bias. **(C)** Egger’s test included studies on MBI.

Sensitivity analysis was performed after sequentially excluding each study, and the recalculated summary results did not change significantly, indicating that no peripheral studies significantly affected the overall results (Figures 12A, 13A, 14A). Funnel plots and Egger’s test were used to assess publication bias based on the FMA-UE, MSA, and MBI scales. The distribution of the funnel plot (Figures 12B, 14B) was asymmetrical. Egger’s test showed FMA-UE (*p* = 0.005; Figure 12C), MSA (*p* = 0.047; Figure 13B), and MBI (*p* = 0.008; Figure 14C), suggesting that publication bias is difficult to rule out.

#### 3.5.6 Quality of evidence

The GRADE approach was employed to assess the quality of evidence from 16 RCTs (Table 3). The analysis focused on three outcome measurements: FMA-UE improvement, MAS improvement, and MBI improvement. Overall, the quality of evidence was ranked from low to very low. The results indicated that none of the outcomes were supported by high-quality evidence; two out of three (2/3, 66.67%) outcomes had low-quality evidence, while one out of three outcomes (1/3, 33.33%) had very low-quality evidence. The main reasons for the low-quality ratings were risk of bias (ROB) and inconsistency.

## 4 Discussion

### 4.1 Summary of main findings

This meta-analysis included 16 RCTs involving 1,218 stroke patients and was conducted to investigate TEAS to the influence of the upper limb functional recovery after stroke. The findings from the meta-analysis indicate that TEAS can significantly improve upper limb motor function, spasticity, and functional independence in stroke patients compared with the control group. The results are consistent with previous research results (Tang et al., 2021). However, considering the literature, the overall quality is low, with issues such as lack of follow-up, absence of adverse reaction reports, and the case fatality rate affecting the credibility. According to the GRADE guidelines, the recommended level is “very low” to “low.” This result is not very convincing, requiring a larger sample size and further evidence. In addition, the meta-analysis showed that the *I^2^* of FMA-UE, MAS, and MBI were all greater than 50%, indicating high heterogeneity among trials. To improve the reliability of the conclusions, subgroup analysis based on control interventions and TEAS parameters (including frequency, treatment duration, and retention time) was performed to find potential sources of heterogeneity.

The trials included in this meta-analysis had differences in the control group intervention, and the treatment measures of the control group included traditional rehabilitation therapies such as neurodevelopmental therapy, exercise therapy, and occupational therapy. Traditional rehabilitation therapy is based on the “motor relearning learning theory,” which promotes the reconstruction of brain motor function through repeated purposeful exercise training (Dimyan and Cohen, 2011). Studies have confirmed that stroke patients often have multiple functional disorders, and task-oriented training and sensory stimulation of TEAS are complementary to each other, which can enhance the rehabilitation effect (Zhao et al., 2015). This is consistent with our results that treatment with the addition of TEAS significantly improved FMA-UE, MAS, and MBI scores compared with conventional rehabilitation alone. However, TEAS treatment did not significantly improve FMA and MBI compared with sham TEAS. This negative result is consistent with a previous TEAS study with a similar design (Alwhaibi et al., 2021). The prevalent methods for sham TEAS underwent identical settings and manipulations as those in the TEAS group, with the exception that the wire connecting the electrode pads to the TEAS stimulator was severed, thereby preventing any current from being delivered. Alwhaibi et al. (2021) compared the effects of task-specific training (TST) combined with TEAS (applied to LI4 and LI11) and sham TEAS combined with TST on upper limb movement in patients with chronic stroke. The results showed that patients in both groups improved significantly after treatment. However, when the two groups were compared, there was no significant improvement in the FMA-UE score after treatment. Even though sham TEAS is designed to have no therapeutic effect, it may still produce unintended consequences. Participants might perceive an improvement due to the placebo effect, which refers to the beneficial outcomes generated by an inactive or ineffective treatment. The experience of receiving stimulation, regardless of its sham nature, could lead to habituation or learning effects, potentially influencing participants’ behavior and performance on the FMA-UE and BMI assessments. Furthermore, participants who believe they are undergoing treatment may become more attuned to their movement abilities, which could affect their performance.

TEAS stimulation parameters such as frequency, treatment duration, and retention time may significantly affect the intervention’s efficacy. In terms of TEAS frequency, the 5/6 times per week treatment was superior to the control group in improving FMA-UE and MBI scores. In contrast, the once-a-day treatment was more effective in improving MAS. The possible explanation is that the effect of relieving spasticity is not sustainable due to the gradual weakening of the effector substance with the prolongation of the treatment interval (Yin et al., 2022). Regarding the duration of treatment, TEAS was superior to the control group in improving FMA-UE, MAS, and MBI scores in 0–4 weeks, 5–8 weeks, and more than 8 weeks. However, the improvement effect was most obvious during 0–4 weeks, which may be the most obvious response of the body at this effective stimulation amount. A previous study (Dimitrijević et al., 1972) suggested that regular and continuous electrical stimulation for a long time could lead to stimulation tolerance but could not cause stimulation of the motor area of the cerebral cortex (M1). For the retention time of TEAS, whether less than 30 min or more than 30 min, TEAS was superior to the control group in improving FMA-UE, MAS, and MBI scores. The study of Laddha (Laddha et al., 2015) showed that transcutaneous electrical stimulation for 30 min or 60 min for 3 weeks was effective on spasticity of lower limbs and walking ability, and there was no significant difference in efficacy. However, the lack of standardization of TEAS parameters is the limitation of its clinical treatment, and more RCTs with rigorous designs based on the differences in TEAS parameters are needed to explore the best TEAS regimen.

A total of six acupoints were included across 16 RCTs, with TE5 (Waiguan, used 14 times) and LI10 (Shousanli, used 13 times) being the most common. From the anatomical point of view, the main muscles under TE5 (Waiguan) are the extensor of the little finger, the extensor of the long thumb, and the extensor of the index finger. Stimulation is beneficial for improving the adverse movement of the wrist joint and promoting the extension of the thumb and index finger. The main muscles under the acupoint LI10 (Shousanli) are extensor carpi radialis longus, extensor carpi radialis brevis, and supinator. The forearm supination can be promoted by stimulating the acupoint, and the spasm can be relieved. TEAS can exert the effects of electrical stimulation and acupoints to promote the recovery of limb function after a stroke (Lim et al., 2023; Zhang et al., 2018).

### 4.2 Mechanisms of TEAS

A related study (Wang D. et al., 2023) has shown that the possible mechanism of limb spasticity and motor dysfunction is that the corresponding cortex of the brain is damaged after stroke, blocking its nerve conduction pathway, resulting in the reduction of local cerebral blood flow and the damage of neuromuscular innervation. TEAS can stimulate and contract the paralyzed muscles of limbs so that they can input information impulses to the nerve center, improve the excitability of relevant brain functional nerve areas, and promote the recovery of neuronal function (Li et al., 2022). Ischemia and hypoxia of brain tissue are the pathological basis of ischemic stroke. Studies have shown that TEAS, by adjusting the SIRT1/FOXO3a and SIRT1/BRCC3/NLRP3 signaling pathways following ischemic stroke, inhibits cell apoptosis, oxidative stress, and inflammation of the nerve to reduce brain damage (Tan et al., 2024) and accelerates the recovery of neurons and some functional metabolism (Nelles et al., 2001). In addition, TEAS can inhibit the TLR4/MyD88/NF-κB pathway, reduce ischemic brain damage after stroke, inhibit inflammation, cell death, and microglia activation (Wu et al., 2023), improve the motor neurons in spinal cord downlink control function, and reduce excessive muscle tone. Another study of TEAS in the treatment of upper limb spastic paralysis showed that TEAS had a significant effect on the excitability of motor-evoked potential in the affected cerebral hemisphere of stroke patients, which was speculated to be related to the enhancement of local cerebral cortical excitability through motor and sensory transduction pathways (Zhang et al., 2013).

### 4.3 Strengths and limitations

This review has some strengths. Our study includes the recent trial and comprehensive analysis of TEAS on upper limb function after stroke. We employed sensitivity analysis and subgroup analysis based on the different control groups and TEAS parameters (frequency, duration, and retention time) to investigate the sources of heterogeneity and assess the stability of our findings. In addition, funnel plots and Egger’s test were employed to evaluate the potential for publication bias. However, this review has some limitations. First, the studies included in this analysis were not registered as clinical trials and did not provide details on sample size calculations. Second, the overall methodological quality of all the analyzed studies was low, and allocation concealment and blinding were not described in detail. Two studies had more than 5% missing outcome data, which may have been subject to implementation and measurement biases. Third, only studies published in Chinese and English were included, which may introduce publication bias. Finally, adverse events were rarely reported in the original study, and safety concerns cannot be assured. Therefore, we interpret the results cautiously.

## 5 Conclusion

Existing evidence suggests that TEAS can improve upper limb motor function and spasticity in stroke patients and improve daily life abilities. The improvement in upper limb motor function appears to have a dose-dependent relationship with the frequency, duration, and retention time of the TEAS intervention. However, these findings should be interpreted cautiously due to the restricted number and low methodological quality of the included trials. High-quality, large-sample, multi-center trials are needed to further confirm these preliminary findings.

## Data Availability

The original contributions presented in the study are included in the article/Supplementary material, further inquiries can be directed to the corresponding author.
