# Supplementary material for: Transcutaneous electrical acupoint stimulation for upper limb motor recovery after stroke: a systematic review and meta-analysis
Source: Front Aging Neurosci. 2024 Nov 27;16:1438994. doi: 10.3389/fnagi.2024.1438994 (PMC11631906; doi:10.3389/fnagi.2024.1438994)
Supplement: Supplementary file 1 [file Data_Sheet_1.pdf]

Transcutaneous Electrical Acupoint Stimulation on Upper Limb Motor Recovery After Stroke: A Systematic Review and Meta-Analysis

Supplementary Material: Search strategy

PubMed

Search	Terms
#1	"Transcutaneous electrical acupoint stimulation"[Title/Abstract] OR "TEAS"[Title/Abstract] OR "transcutaneous acupoint electrical stimulation"[Title/Abstract] OR "TAES"[Title/Abstract] OR "acustimulation"[Title/Abstract]
#2	"stroke"[Mesh] OR "apoplexy"[Mesh]
#3	"stroke"[Title/Abstract] OR "Cerebrovascular Accident"[Title/Abstract] OR "CVA"[Title/Abstract] OR "Cerebrovascular Apoplexy"[Title/Abstract] OR "Brain Vascular Accident"[Title/Abstract] OR "Cerebrovascular Stroke"[Title/Abstract] OR "Apoplexy"[Title/Abstract] OR "Cerebral Stroke"[Title/Abstract] OR "Acute Stroke"[Title/Abstract] OR "Acute Cerebrovascular Accident"[Title/Abstract]
#4	#2 OR #3
#5	"Hemiplegia"[MeSH] OR "Paralysis"[MeSH]
#6	"Upper limb"[Title/Abstract] OR "Upper extremity"[Title/Abstract] OR "shoulder"[Title/Abstract] OR "arm"[Title/Abstract] OR "forearm"[Title/Abstract] OR "wrist"[Title/Abstract] OR "hand"[Title/Abstract] OR "finger"[Title/Abstract] OR "motor function"[Title/Abstract]
#7	#5 AND #6
#8	("randomized controlled trial"[Publication Type] OR "controlled clinical trial"[Publication Type] OR "randomized"[Title/Abstract] OR "placebo"[Title/Abstract] OR "clinical trials as topic"[MeSH Terms] OR "randomly"[Title/Abstract] OR "trial"[Title]) NOT ("animals"[MeSH Terms] NOT "humans"[MeSH Terms])
#9	#1 AND #4 AND #7 AND #8

Web of Science

Search	Terms
#1	TS = (Transcutaneous electrical acupoint stimulation OR TEAS OR transcutaneous acupoint electrical stimulation OR TAES OR acustimulation)
#2	TS = (stroke OR Cerebrovascular Accident OR CVA OR Cerebrovascular Apoplexy OR Brain Vascular Accident OR Cerebrovascular Stroke OR Apoplexy OR Cerebral Stroke OR Acute Stroke OR Acute Cerebrovascular Accident)
#3	TS = (Hemiplegia OR Paralysis OR Upper limb OR Upper extremity OR shoulder OR arm OR forearm OR wrist OR hand OR finger OR motor function)
#4	TS = (randomized OR randomly OR placebo OR trial)
#5	#1 AND #2 AND #3 AND #4

The Cochrane Library

Search	Terms
#1	(Transcutaneous electrical acupoint stimulation):ti,ab,kw OR (TEAS):ti,ab,kw OR (transcutaneous acupoint electrical stimulation):ti,ab,kw OR (TAES):ti,ab,kw OR (acustimulation):ti,ab,kw
#2	(stroke):ti,ab,kw OR (Cerebrovascular Accident):ti,ab,kw OR (CVA):ti,ab,kw OR (Cerebrovascular Apoplexy):ti,ab,kw OR (Brain Vascular Accident):ti,ab,kw OR (Cerebrovascular Stroke):ti,ab,kw OR (Apoplexy):ti,ab,kw OR (Cerebral Stroke):ti,ab,kw OR (Acute Stroke):ti,ab,kw OR (Acute Cerebrovascular Accident):ti,ab,kw
#3	(Hemiplegia):ti,ab,kw OR (Paralysis):ti,ab,kw OR (Upper limb):ti,ab,kw OR (Upper extremity):ti,ab,kw OR (shoulder):ti,ab,kw OR (arm):ti,ab,kw OR (forearm):ti,ab,kw OR (wrist):ti,ab,kw OR (hand):ti,ab,kw OR (finger):ti,ab,kw OR (motor function):ti,ab,kw
#4	(randomized):ti,ab,kw OR (randomly):ti,ab,kw OR (placebo):ti,ab,kw OR (tiral):ti,ab,kw
#5	#1 AND #2 AND #3 AND #4

Embase

Search	Terms
#1	'Transcutaneous electrical acupoint stimulation':ab,ti OR 'TEAS':ab,ti OR 'transcutaneous acupoint electrical stimulation':ab,ti OR 'TAES':ab,ti OR 'acustimulation':ab,ti
#2	'stroke':ab,ti OR 'Cerebrovascular Accident':ab,ti OR 'CVA':ab,ti OR 'Cerebrovascular Apoplexy':ab,ti OR 'Brain Vascular Accident':ab,ti OR 'Cerebrovascular Stroke':ab,ti OR 'Apoplexy':ab,ti OR 'Cerebral Stroke':ab,ti OR 'Acute Stroke':ab,ti OR 'Acute Cerebrovascular Accident':ab,ti
#3	'Hemiplegia':ab,ti OR 'Paralysis':ab,ti OR 'Upper limb':ab,ti OR 'Upper extremity':ab,ti OR 'shoulder':ab,ti OR 'arm':ab,ti OR 'forearm':ab,ti OR 'wrist':ab,ti OR 'hand':ab,ti OR 'finger':ab,ti OR 'motor function':ab,ti
#4	('randomized controlled trial':ab,ti OR ('controlled clinical trial' OR 'randomized':ab,ti OR 'randomly':ab,ti OR 'trial':ab,ti OR 'placebo':ab,ti OR 'clinical article':ab,ti OR 'clinical trial':ab,ti OR 'controlled study':ab,ti OR 'major clinical study':ab,ti OR 'double blind procedure':ab,ti OR 'multicenter study':ab,ti OR 'single blind procedure':ab,ti OR 'crossover procedure':ab,ti))
#5	#1 AND #2 AND #3 AND #4

China National Knowledge Infrastructure （CNKI）

Search	Terms
#1	(SU = '经皮穴位电刺激' OR SU = '穴位电刺激' OR SU = '经皮电刺激' OR SU = 'TEAS')
#2	(SU = '脑卒中' OR SU = '中风' OR SU = '偏瘫' OR SU = '脑血管意外' OR SU = '脑梗死' OR SU = '脑出血')
#3	(SU = '上肢功能' OR SU = '手功能')
#4	(SU=随机 OR FT=随机)
#5	#1 AND #2 AND #3 AND #4

Database for Chinese Technical Periodicals (VIP)

Search	Terms
#1	M=（经皮穴位电刺激 OR 穴位电刺激 OR 经皮电刺激 OR TEAS）
#2	M=（OR 中风 OR 偏瘫 OR 脑血管意外 OR 脑梗死 OR 脑出血）
#3	M=（上肢功能 OR 手功能）
#4	M=（试验 OR 观察 OR 随机 OR 疗效评价）
#5	#1 AND #2 AND #3 AND #4

Wanfang Database

Search	Terms
#1	（经皮穴位电刺激 OR 穴位电刺激 OR 经皮电刺激 OR TEAS）
#2	（脑卒中 OR 中风 OR 偏瘫 OR 脑血管意外 OR 脑梗死 OR 脑出血）
#3	（上肢功能 OR 手功能）
#4	（试验 OR 观察 OR 随机 OR 疗效评价）
#5	#1 AND #2 AND #3 AND #4

Chinese Biomedical Literature Database

Search	Terms
#1	（经皮穴位电刺激 OR 穴位电刺激 OR 经皮电刺激 OR TEAS）
#2	（脑卒中 OR 中风 OR 偏瘫 OR 脑血管意外 OR 脑梗死 OR 脑出血）
#3	（上肢功能 OR 手功能）
#4	（试验 OR 观察 OR 随机 OR 疗效评价）
#5	#1 AND #2 AND #3 AND #4
